# Supplementary material for: Colloidal interactions and unusual crystallization versus de-mixing of elastic multipoles formed by gold mesoflowers
Source: Nat Commun. 2020 Jan 10;11:188. doi: 10.1038/s41467-019-14031-2 (PMC6954209; doi:10.1038/s41467-019-14031-2)
Supplement: Supplementary file 1 — Supplementary Information [file 41467_2019_14031_MOESM1_ESM.pdf]

Supporting Information

Colloidal interactions and unusual crystallization versus de-mixing of elastic multipoles formed by gold mesoflowers

Ye Yuan,[1] Mykola Tasinkevych[2,3] & Ivan I. Smalyukh[1,4,5*]

[1]*Department of Physics and Soft Materials Research Center, University of Colorado, Boulder, CO 80309, USA*

[2]*Departamento de Física, Faculdade de Ciências, Universidade de Lisboa, Campo Grande P-1749-016 Lisboa, Portugal*

[3]*Centro de Física Teórica e Computacional, Universidade de Lisboa, Campo Grande P-1749-016 Lisboa, Portugal*

[4]*Department of Electrical, Computer, and Energy Engineering, Materials Science and Engineering Program, University of Colorado, Boulder, CO 80309, USA*

[5]*Renewable and Sustainable Energy Institute, National Renewable Energy Laboratory and University of Colorado, Boulder, CO 80309, USA*

**Email: ivan.smalyukh@colorado.edu*

Supplymentary Figures:

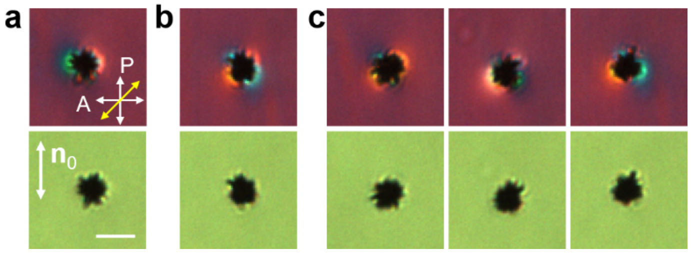

Supplementary Fig. 1 | A single mesoflower embedded in the LC showing different metastable elastic multipoles. a-c, Polarizing optical micrographs of the same gold mesoflower in metastable states with dipole-like (**a**), quadrupole-like (**b**) and even more complex (**c**) higher-order multipolar director distortions. The far-field alignment along $\mathbf{n}_0$ is indicated by the white double arrow; P and A show the crossed polarizations of the polarizer and analyzer, respectively; yellow double arrow shows the slow axis of a 530 nm retardation plate inserted between the polarizers. Scale bar is 3 μm.

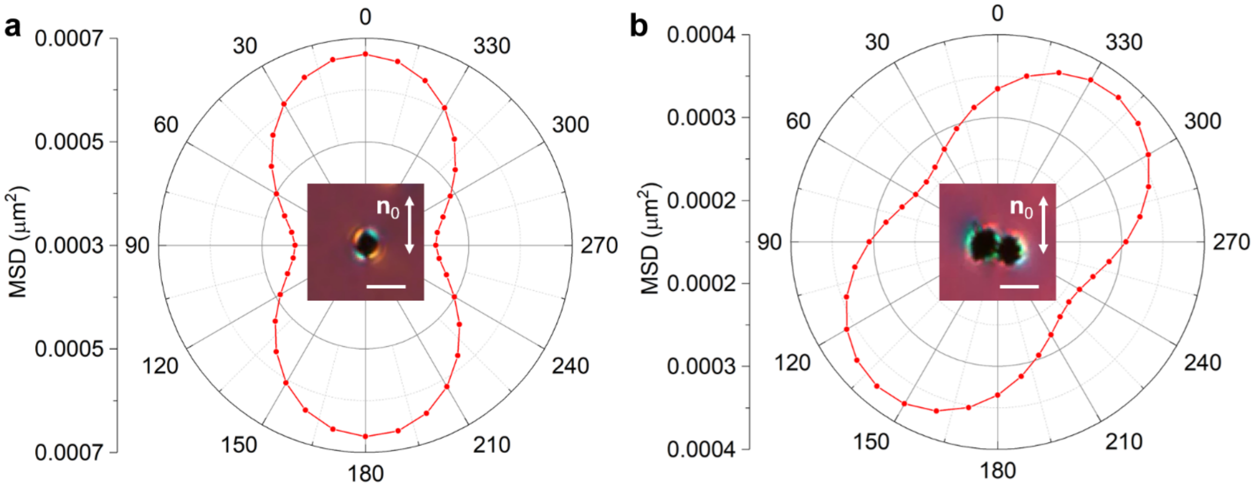

Supplementary Fig. 2 | Anisotropic Brownian motion of colloidal particles in an aligned LC. **a**, Angular-dependent MSD of a gold colloidal sphere. **b**, Angular-dependent MSD of an assembly consisting of two mesoflowers. Insets show corresponding polarizing optical micrographs of the studied particles. Images are taken under crossed polarizers with a 530 nm retardation plate; white double arrows indicate $\mathbf{n}_0$. Scale bars are 3 μm.

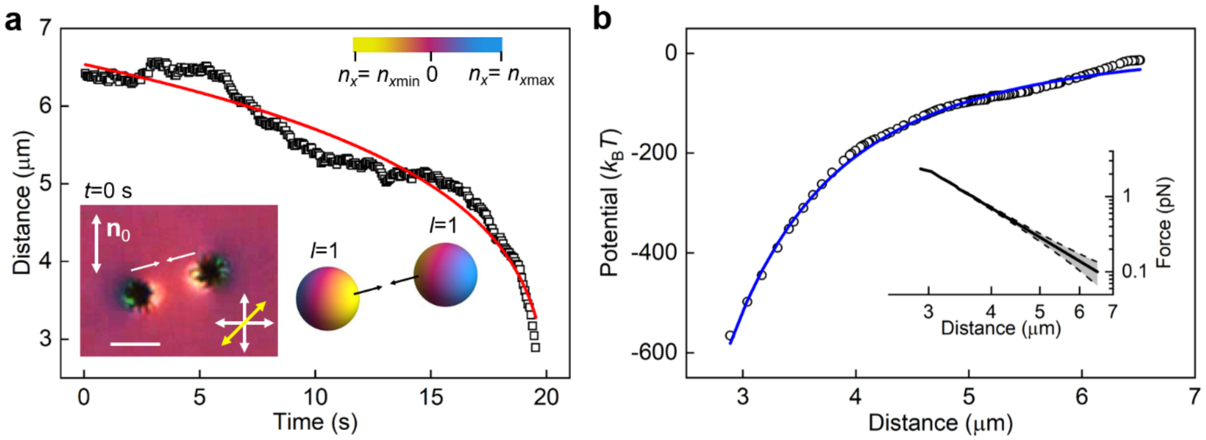

Supplementary Fig. 3 | Attractive dipole-dipole interaction between mesoflowers. a, Separation distance versus time for attraction between two mesoflowers inducing dipole-like structures of director field. The micrograph inset shows the initial position states of particles set by laser tweezer under polarizing optical microscopy. Schematics in the insets are visualization of the x-component of $\mathbf{n}(\mathbf{r})$ on the spherical surface enclosing two dipoles of opposite sign. The direction of interaction is shown with pairs of arrows. The red curve is the best fit of the experimental data with the function $r_c(t)=(r_0^n\text{-}n\alpha t)^{1/n}$, where n=5 for the dipole-dipole interaction; the fitting coefficients are r_0=6.5 μm and α=1.2×10^2 μm^5 s^{-1}. **b**, Interaction potential versus distance corresponding to **a** with inset showing distance dependence of force plotted using the log-log scale. The blue curve is the best fit with a power-law function $\propto$-r_c^{-3} corresponding to dipole-dipole interaction potential, from which the force is calculated. Grey bands with dashes in the inset represent estimated error of the force. Scale bar in the inset is 3 μm.

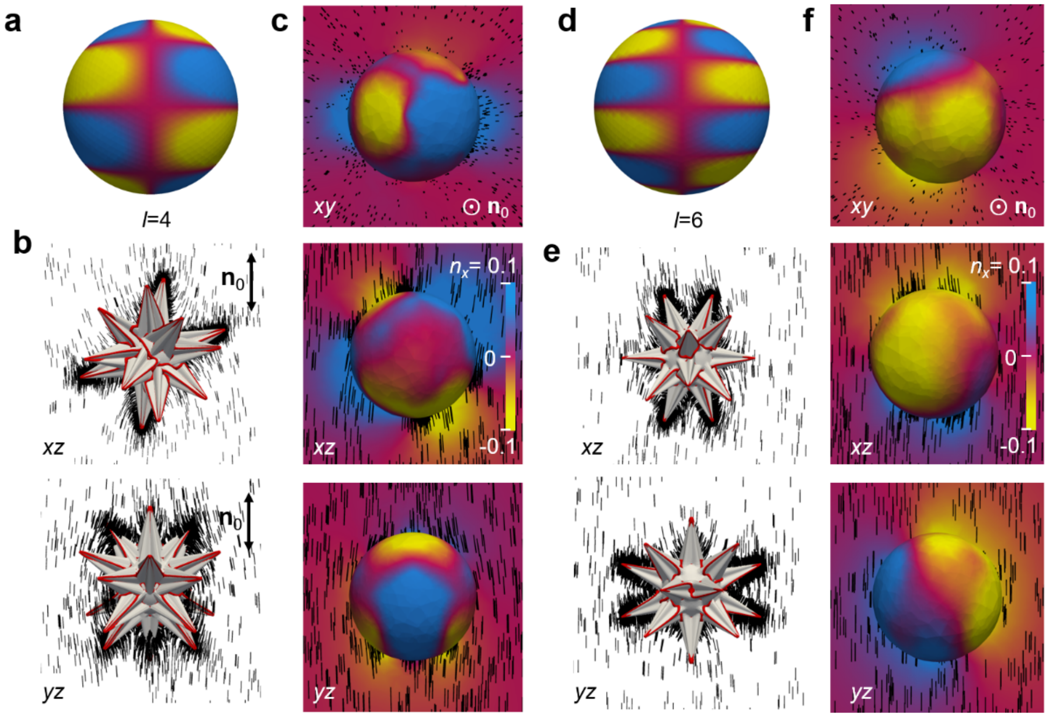

Supplementary Fig. 4 | Hexadecapole and 64-pole. a, **d**, Hexadecapole and 64-pole, respectively, around spherical particles with color-coded diagrams of the x-component n_x of the director field. **b**, **e**, Director structure around a mesoflower with dominant elastic hexadepole (**b**) and 64-pole (**e**) contribution. The director fields are shown by rods and defect lines depicted as red tubes. **c, f**, Color-coded diagrams of n_x in the xy, xz, and yz cross sections as well as at the interpolation spheres around the mesoflower with the dominant eleastice hexadecapole (**c**) and 64-pole (**f**) contribution.

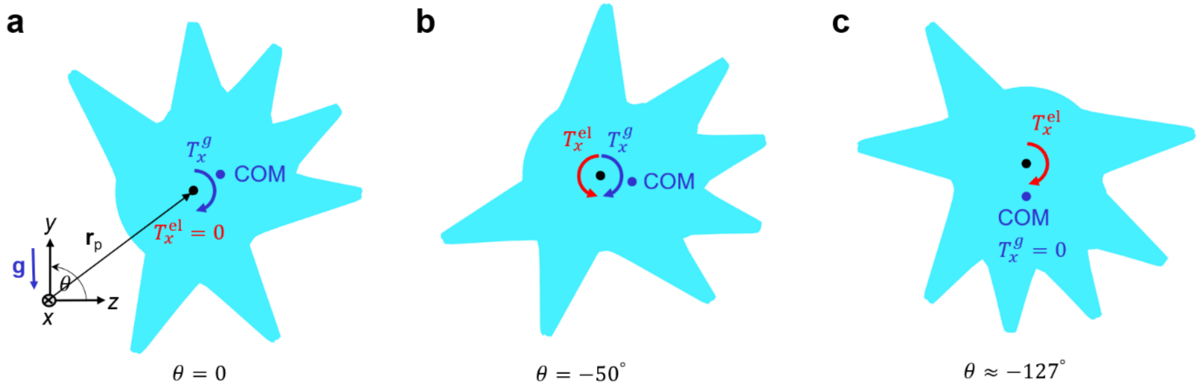

Supplementary Fig. 5 | Cross sections of the mesoflower in Fig. 5c with a dominant elastic monopole. a, The mesoflower at the orientation with vanishing x-component, $T_x^{\mathrm{el}} = 0$, of the elastic torque about the center $\mathbf{r}_\mathrm{p}$ of the spherical core of the particle, shown by the solid black dot. The x-component T_x^g of the gravity torque rotates the particle clockwise (see the blue curve in Fig.5c). Solid blue dot represents the center of mass (COM) of the particle; **g** depicts the direction of the gravitational acceleration. **b**, The mesoflower is tilted away from the elastic equlibrium. At this orientation T_x^{el} and T_x^g act in the oposite directions, conterclockwise and clockwise, respectively. **c**, The particle orientation is such that the particle center of mass and the torque pivot point $\mathbf{r}_\mathrm{p}$ are on the y axis, which renders $T_x^g = 0$. T_x^{el} here rotates the particle clockwise (Fig. 5c).

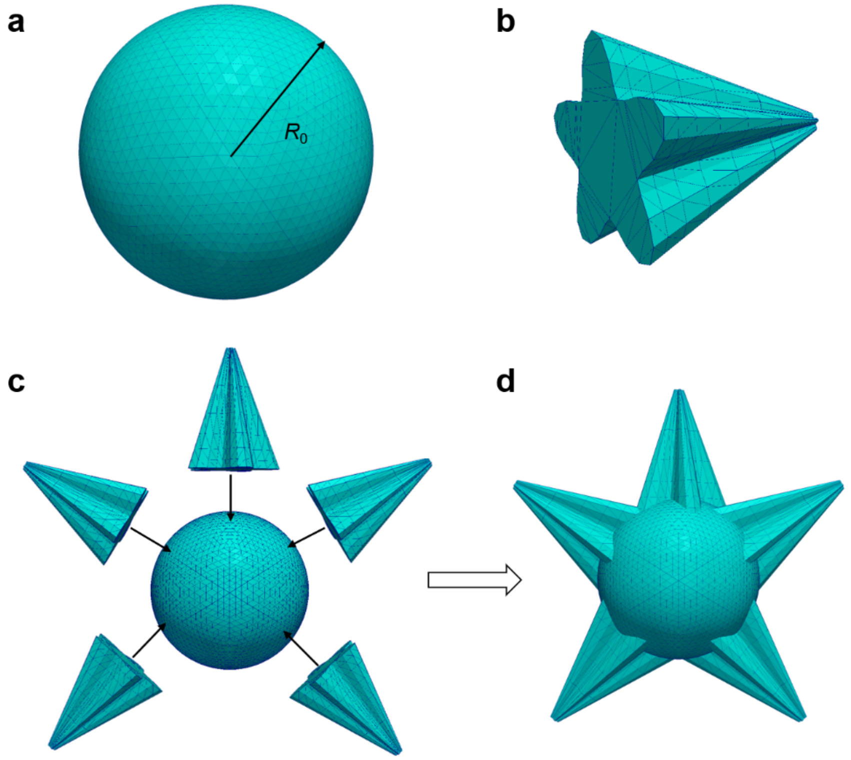

Supplementary Fig. 6 | Schematics of the numerical generation of a mesoflower with five spikes. **a**, A triangulated surface of a sphere which will be the core of the mesoflower. The sphere is characterized by its radius R_0. **b**, A triangulated surface of one of the spikes of the intended mesoflower. **c**, Five spikes are placed at the predefined orientations $\mathbf{\Omega}_i, i = 1,..,5,$ relative to the core of the planned mesoflower. **d**, Each of the spikes is translated along its $\mathbf{\Omega}_i$ towards the core and then merged with the core, by using the "*union*" function of the Gnu Triangulated Surface library, which results in a triangulated surface of the five-spike mesoflower. Other mesoflowers used in the numerical simulation are generated in a similar way with the systematically varied number and orientations of spikes.

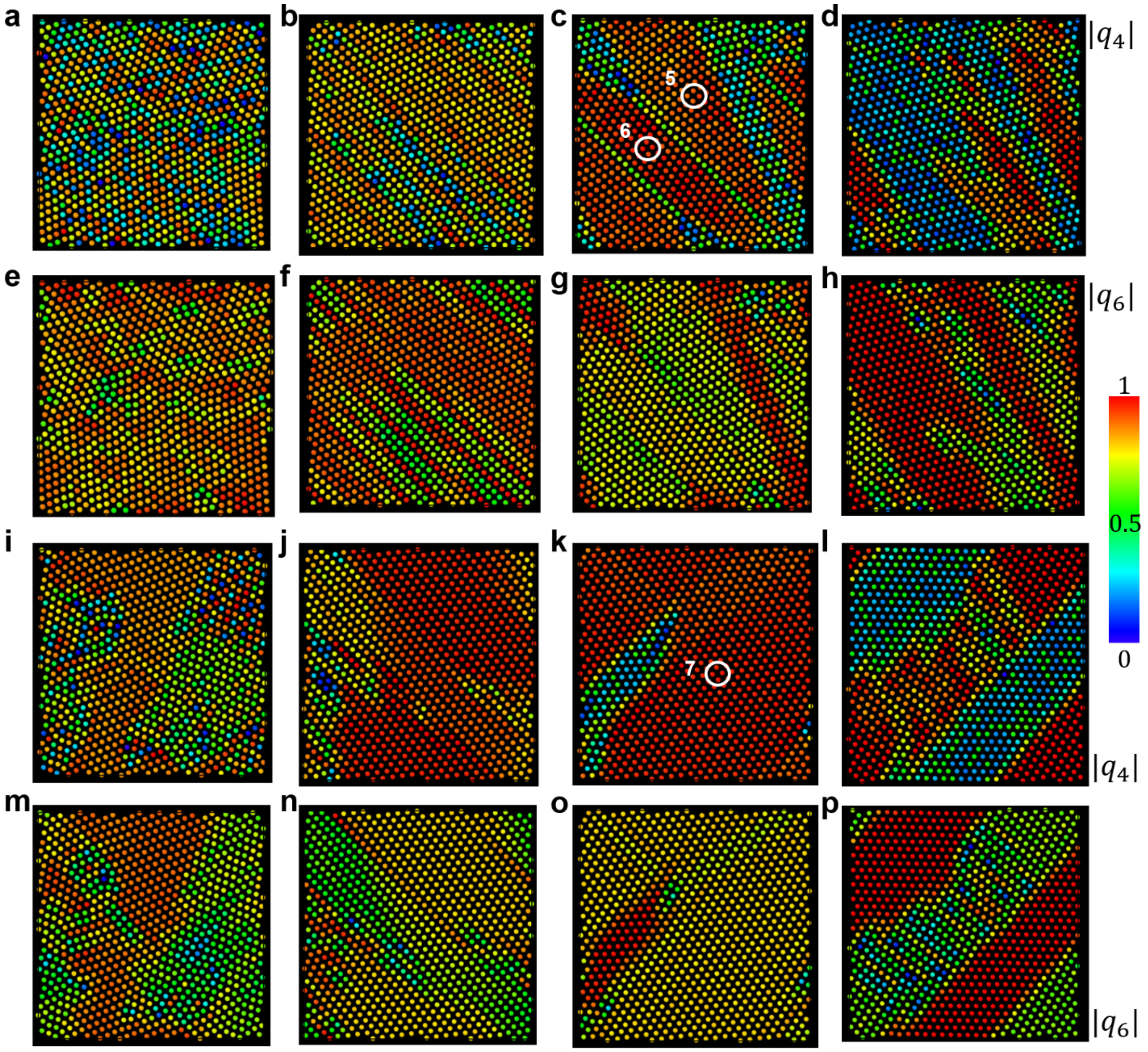

Supplementary Fig. 7 | Self-organization of hexadecapole colloids with stronger Yukawa repulsion. a-h, Snapshots of temporal evolution of self-assembled structures. Panels in the same column are taken at the same time during the simulation; time elapses from (**a**) to (**d**). Spheres in the top (bottom) row are colored according to the absolute value of the local quartic order parameter $q_4(j)$ (hexatic order parameter $q_6(j)$) as marked on the right with the color scale as an inset. Parameters used are $Q_4 = 3 \times 10^{-5}$, $\frac{r_c}{R_{\text{eff}}} = 5$, $\kappa R_{\text{eff}} = 0.3$, $\frac{A}{\bar{K} R_{\text{eff}}^2} = 1$. The system size $L_x \times L_y = 34 \times 34\ R_{\text{eff}}^2$ in (**a**), (**e**); $L_x \times L_y = 35 \times 35\ R_{\text{eff}}^2$ in (**b**), (**f**); $L_x \times L_y = 36 \times 36\ R_{\text{eff}}^2$ in (**c**), (**g**); and $L_x \times L_y = 37 \times 37\ R_{\text{eff}}^2$ in (**d**), (**h**). **i-p**, Panels are arranged and colored in the same way as (**a**)-(**h**). Parameters used are $Q_4 = 3 \times 10^{-4}$, $\frac{r_c}{R_{\text{eff}}} = 5$, $\kappa R_{\text{eff}} = 0.3$, $\frac{A}{\bar{K} R_{\text{eff}}^2} = 10$. The system size $L_x \times L_y = 34 \times 34\ R_{\text{eff}}^2$ in (**i**), (**m**); $L_x \times L_y = 35.8 \times 35.8\ R_{\text{eff}}^2$ in (**j**), (**n**); $L_x \times L_y = 36 \times 36\ R_{\text{eff}}^2$ in (**k**), (**o**); and $L_x \times L_y = 37 \times 37\ R_{\text{eff}}^2$ in (**l**), (**p**). The size of the spheres in all panels is shown not to scale.
