# Supplementary material for: Colloidal interactions and unusual crystallization versus de-mixing of elastic multipoles formed by gold mesoflowers
Source: Nat Commun. 2020 Jan 10;11:188. doi: 10.1038/s41467-019-14031-2 (PMC6954209; doi:10.1038/s41467-019-14031-2)
Supplement: Supplementary file 2 — Description of Additional Supplementary Files [file 41467_2019_14031_MOESM2_ESM.pdf]

Description of Additional Supplementary Files

Supplementary Movie 1 | Segregation of a binary mixture of dissimilar elastic monopoles. The red (blue) spheres represent positively (negatively) charged elastic monopoles. Initial condition is set to be NaCl-like square lattices. Over time, the homogenous mixture separates into distinctive regions where like-charged monopoles aggregate. The video corresponds to Fig. 8.

Supplementary Movie 2 | Spontaneous crystallization of elastic hexadecapoles with grain boundaries. The system starts with a glassy state and evolves into a state with grain boundaries between regions of different lattice orientations. The spheres are colored according to the strength of local quadratic order parameter q_4; red indicates $|q_4|$~1, and blue for $|q_4|$~0. See the inset of Fig. 9b for the color scale bar. The video corresponds to Fig. 9c-e.

Supplementary Movie 3 | Self-assembly of hexadecapole colloids with longer-stronger Yukawa repulsion. Different rhombic lattices emerge by varying the strength of hexadecapolar moment and Yukawa repulsion. Parameters used are $Q_4 = 9 \times 10^{-5}$, $\frac{A}{\bar{K}R_{\text{eff}}^2} = 1$. The spheres are colored according to the strength of local quadratic order parameter q_4; red indicates $|q_4|$~1, and blue for $|q_4|$~0. See the inset of Fig. 10 for the color scale bar. The video corresponds to Fig. 10a-d.

Supplementary Movie 4 | Self-assembly of hexadecapole colloids with longer-stronger Yukawa repulsion. Different rhombic lattices emerge by varying the strength of hexadecapolar moment and Yukawa repulsion. Parameters used are $Q_4 = 9 \times 10^{-5}$, $\frac{A}{\bar{K}R_{\text{eff}}^2} = 10$. The spheres are colored according to the strength of local quadratic order parameter q_4; red indicates $|q_4|$~1, and blue for $|q_4|$~0. See the inset of Fig. 10 for the color scale bar. The video corresponds to Fig. 10i-l.

Supplementary Movie 5 | Self-assembly of hexadecapole colloids with longer-stronger Yukawa repulsion. Different rhombic lattices emerge by varying the strength of hexadecapolar moment and Yukawa repulsion. Parameters used are $Q_4 = 3 \times 10^{-5}$, $\frac{A}{\bar{K}R_{\text{eff}}^2} = 1$. The spheres are colored according to the strength of local quadratic order parameter q_4; red indicates $|q_4|$~1, and blue for $|q_4|$~0. See the inset of Supplementary Fig. 7 for the color scale bar. The video corresponds to Supplementary Fig. 7a-d.

Supplementary Movie 6 | Self-assembly of hexadecapole colloids with longer-stronger Yukawa repulsion. Different rhombic lattices emerge by varying the strength of hexadecapolar moment and Yukawa repulsion. Parameters used are $Q_4 = 3 \times 10^{-4}$, $\frac{A}{\bar{K}R_{\text{eff}}^2} = 10$. The spheres are colored according to the strength of local quadratic order parameter q_4; red indicates $|q_4|$~1, and blue for $|q_4|$~0. See the inset of Supplementary Fig. 7 for the color scale bar. The video corresponds to Supplementary Fig. 7i-l.
